# Maximising recruitment of research participants into a general practice based randomised controlled trial concerning lung diagnosis—staff insights from an embedded qualitative study

**DOI:** 10.1186/s13063-022-06125-y

**Published:** 2022-03-21

**Authors:** Hayley Prout, Angela Tod, Richard Neal, Annmarie Nelson

**Affiliations:** 1 Centre for Trials Research (CTR), College of Biomedical & Life Sciences, Cardiff University, 4th Floor, Neuadd Meirionnydd, Heath Park, Cardiff, CF14 4YS UK; 2 Division of Nursing and Midwifery, The University of Sheffield, Barber House Annex 3a Clarkehouse Rd, Sheffield, S10 2LA UK; 3 University of Exeter Medical School, St Luke’s Campus, 1.12 College House, Magdalen Road, Exeter, EX1 2LU UK; 4 Marie Curie Palliative Care Research Centre, School of Medicine, Cardiff University, 8th Floor Neuadd Meirionydd, Heath Park, Cardiff, CF14 4YS UK

## Abstract

**Background:**

The ELCID Trial was a feasibility randomised controlled trial examining the effect on lung cancer diagnosis of lowering the threshold for referral for urgent chest X-ray for smokers and recent ex-smokers, aged over 60 with new chest symptoms. The qualitative component aimed to explore the feasibility of individually randomising patients to an urgent chest X-ray or not and to investigate any barriers to patient recruitment and participation. This would inform the design of any future definitive trial. This paper explores general practice staff insights into participating in and recruiting to diagnostic trials for possible/suspected lung cancer.

**Methods:**

Qualitative interviews were conducted with 11 general practice staff which included general practitioners, a nurse practitioner, research nurses and practice managers.

Interviews were analysed using a framework approach.

**Results:**

Findings highlight general practice staff motivators to participate in the trial as recruiters, practice staff interactions with patients recruited onto the study, methods of organisation staff used to undertake the trial, the general impact of the trial on practice staff, how the trial research team supported the practices and lastly practice staff suggestions for trial delivery improvement.

**Conclusions:**

The integration of a qualitative component focused on staff experiences participating in a lung diagnostic trial has demonstrated the feasibility to recruit for similar future studies within general practice. Although recruitment into trials can be difficult, results from our study offer suggestions on maximising patient recruitment not just to trials in general but also specifically for a lung diagnosis study.

**Trial registration:**

ClinicalTrials.gov, NCT01344005. Registered on 27 April 2011

## Background

### Recruitment in general practice clinical trials

The importance of research in general practice (GP) is longstanding

The values of general practice underpin a challenging research agenda spanning: preventive medicine, early diagnosis, acute and chronic disease management, personalised care, and the understanding of beliefs and behaviours relating to health and illness. These areas of focus are of increasing importance to the UK's healthcare agenda which promotes healthy living and pro-active disease management. p. 5 [1].

In 2006, the Royal College of General Practitioners (RCGP) set up its Research Ready Scheme to support general practices to be research active and this scheme has, to date, accredited over 500 UK practices [2]. By 2018/2019, the Primary Care Specialty recruited 160,000 participants into the National Institute of Healthcare Research (NIHR) Clinical Research Network studies [3].

Maximising recruitment of research participants into randomised controlled trials (RCTs) within general practice is of great importance if rigorous research is to be carried out. However, the failure to recruit adequate numbers of participants has long been a major barrier to the completion of RCTs in primary care globally [4–6].

The use of qualitative research methods along trials is being increasingly used to bring to light the reasons for recruitment difficulties in trials. In their systematic review of improving the recruitment activity of clinicians in randomised controlled trials, Fletcher and colleagues [7] highlighted the importance of using qualitative research methods alongside trials

‘the most promising intervention identified by this review was the use of qualitative methods embedded in host RCTs to define appropriate methods, targeted at clinicians, relevant to the context of the individual studies’ p.1

Indeed, primary care trials are increasingly using embedded qualitative research methods to uncover important information relating to recruitment onto trials.

### The benefits of embedding qualitative research into primary care randomised controlled trials to explore recruitment issues

Donovan and colleagues [8] used in-depth interviews alongside the ProtecT (prostate testing for cancer and treatment) trial carried out on general practice sites to investigate recruitment challenges between recruiting sites and over time. They found that recruiters had difficulty discussing equipoise and presenting treatments equally and that the terminology that recruiters used was also misinterpreted by participants. This information allowed for changes to be made to the content and presentation of the information and ultimately helped to improve the differing levels of recruitment.

Qualitative interviews were also used with trial staff and recruiting clinicians to explore under recruitment of patients to a community trial concerning patients with severe mental illness and supported employment [9]. Reasons included misconceptions about the trial, a perceived lack of study equipoise, misunderstanding of the trial arms and differing interpretations of eligibility and paternalism. Based on this information, the authors advocate clinician and patient involvement in the study design to improve recruitment in future similar trials.

Paramisivan and colleagues [10] also explored reasons for low recruitment during their qualitative recruitment investigation in the SPARE (Selective bladder Preservation Against Radical Excision) feasibility trial. They highlighted problems relating to equipoise, highlighting treatment preferences amongst both participants and staff. They also found that clinicians had difficulties in identifying eligible patients. Trial information was consequently simplified, recruitment pathway focused around lead recruiters, and training sessions were provided for recruiters. Problems with patient eligibility however could not be resolved.

Similarly, Noble and colleagues [11] illustrated important qualitative research findings relating to patient recruitment difficulties. This trial aimed to identify the most clinically and cost-effective length of anticoagulation with low molecular weight heparin (LMWH) in the treatment of cancer associated thrombosis. Although the study could not recruit adequate numbers for the study, interviews with recruiting clinicians highlighted existing beliefs about medication and the length of time patients needed to be anticoagulated. Study equipoise was also questioned. The authors conclude that the lessons learnt from this study offer useful insights pertaining to the design of future similar studies.

### The ELCID trial

**Fig. 1:**
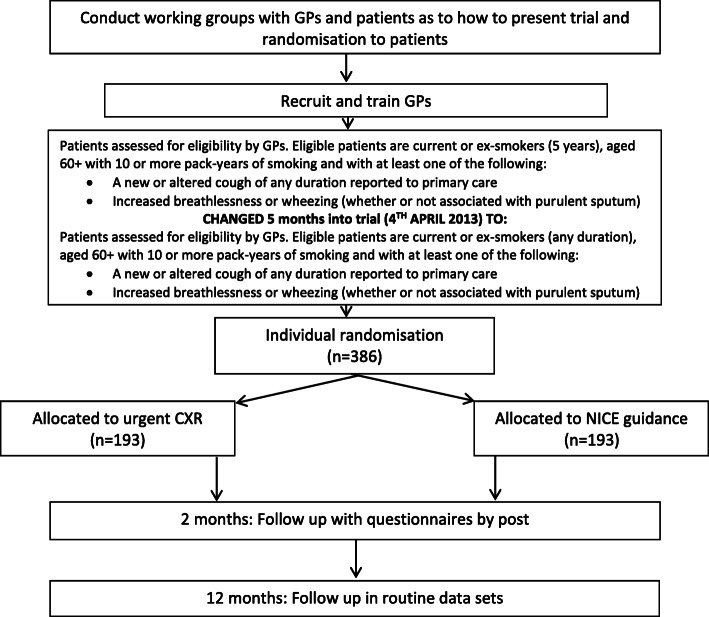
ELCID trial format

The ELCID feasibility trial [12] (Fig. 1) aimed to improve lung diagnosis by examining the value of lowering the threshold for ordering a chest X-ray for suspected lung cancer symptoms in the primary care setting. Specific outcomes included evaluating trial design, materials, and intervention and the training and recruitment of practices, including the recruitment and randomisation of patients.

The control group reflected NICE referral guidance (at the time) whereby patients were urgently referred if they were experiencing one of a number of chest symptoms present for more than 3 weeks. The trial intervention, termed ‘Extra-Nice’, meant randomised patients received an urgent chest X-ray if they presented with one of a number of chest symptoms of any duration, smoked, or were ex-smokers, and who were over 60 years. Eligibility included patients who were over 60 years of age and were either smokers or ex-smokers with 10 or more pack years of smoking history. They also needed to have presented at a general practice with a new or altered cough of any duration or increased breathlessness or wheezing (whether or not associated with purulent sputum) [13]. In view of recruitment difficulties, however, the eligibility status was revised to be less strict and the criteria changed from receiving only current smokers and non-smokers of 5 years or less to accepting smokers and non-smokers with no time duration associated.

The aim of this trial was to inform the design of a large UK-wide, clinical trial [12] to lower the threshold for investigating patients presenting with symptoms of possible lung cancer. The study involved health economics, quality of life, qualitative and quantitative methods, in order to fully assess feasibility.

### The ELCID qualitative study

The integrated qualitative study was carried out with the aim of exploring the feasibility of individually randomising patients to an urgent chest X-ray or not and to investigate any barriers to patient recruitment and participation. A previous paper reported on patient experiences of participating in the trial [14]. This current paper reports on practice staffs’ experiences of participating in the trial. Our paper is the only one to date which has qualitatively explored practice staffs’ experiences of recruiting eligible patients into trials for lung disease diagnosis.

This ELCID qualitative Study is reported in line with the guidelines set out in Consolidated Criteria for Reporting Qualitative Research (COREQ) [15].

### The aim of the paper

This paper aims to identify the critical enablers and barriers to recruitment to a lung disease randomised clinical trial that recruited participants through general practices (GP). It reports and discusses selected findings from the ELCID feasibility trial, a diagnostic trial of lung disease in general practice which examines staff attitudes and experiences of participating in a trial of this nature. The paper focuses on experiences of the staff working in general practices who recruited to ELCID. It addresses a gap in evidence regarding factors influencing recruitment to research in general practice. Recommendations from this qualitative study can be used for the design of the further lung diagnostic trials.

## Methods

### Study design

This was a multicentre, qualitative study which was embedded within a trial.

### Recruitment of GP Practices onto the trial

**Fig. 2:**
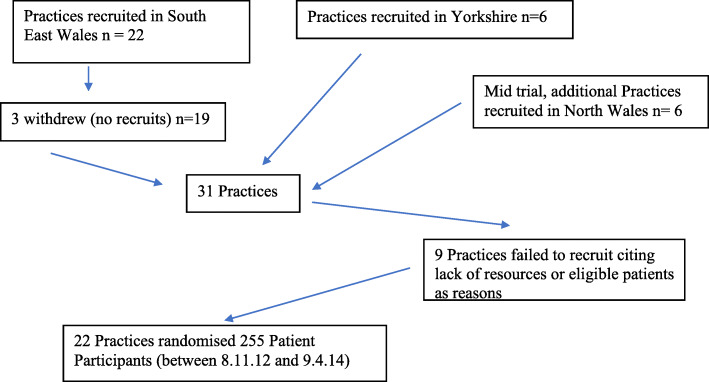
Recruitment of GP practices for ELCID trial

GP practices were recruited into the trial from Wales and Yorkshire with 22 practices ultimately recruiting and randomising 255 patients (Fig. 2). Recruitment of practices was assessed by practice size, known research activity—Primary Care Research Incentive Scheme (PiCRIS) and number of months open to recruitment [12]. Those practices who agreed to take part in the trial also agreed to participate in the embedded qualitative study.

Practices were initially sent a letter to determine their interest in the trial. If they agreed to participate in the trial, the research team would deliver a training day at the practice site which consisted of a PowerPoint presentation covering all aspects of the study.

### Staff roles in the trial

**Fig. 3:**
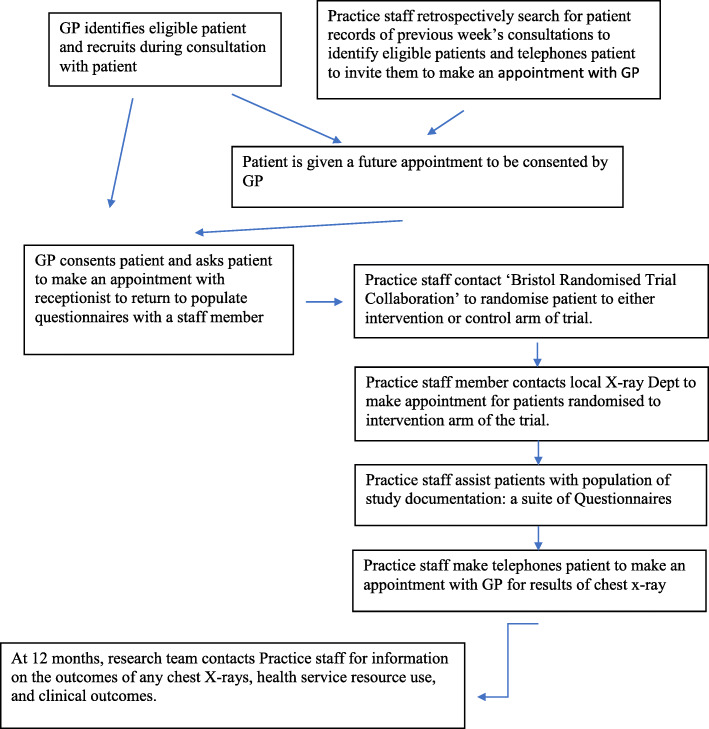
Staff roles and responsibilities

Practice staff had individual roles in the study (Fig. 3). GPs recruited eligible patients during patient consultations and discussed the study with patients and provided written informed consent in the form of participant information sheets and consent forms. If patients were interested in participating, they were either consented to the trial or if they preferred, given a future appointment with the GP to consent.

Practice staff also retrospectively searched patient records of previous week’s consultations (where practice resources allowed) to determine eligible patients. These patients were then telephoned by the practice staff and invited to make an appointment with their GP to discuss the study.

When patients were consented onto the study, they were then individually randomised to either an urgent chest X-ray or usual care (NICE 2015). Randomisation took place centrally via the Bristol Randomised Trials Collaboration. Patients were then informed of their allocation and directed to a practice staff member who assisted them with populating the study documentation, a suite of health questionnaires [16–22] (Table 1). These same questionnaires were also posted to patients 2 months later. Twelve months after the patients’ randomisation, general practices were contacted by the research team for information on the outcomes of any chest X-rays, health service resource use and clinical outcomes.

**Table 1:** Health questionnaires—post randomisation and 2 months

• Hospital Anxiety and Depression Scale (HADS) (Zigmond and Snaith, 1983) • Client Service Receipt Inventory (CSRI) (Beecham and Knapp, 1999; Ridyard and Hughes, 2010; Ridyard et al, 2012 • ICEpop CAPability measure for Older people (ICECAP-O) (Coast et al, 2008) • EuroQol 5 Dimensional Health State Questionnaire (EQ-5D-3 L) (EuroQol Group, 1990; EuroQoL, 2015)	

Practices were reimbursed financially for the time staff spent participating in the trial. Also, occasional prizes were awarded to practices for best or most improved recruitment. During the study, the practices also received newsletters which highlighted information on on-going recruitment between the practices.

### Recruitment of practice staff for qualitative interviews and data collection

During the implementation of the trial, practice managers invited staff to volunteer for an interview with the qualitative researcher. The qualitative researcher (HP) introduced herself to the interviewees by visiting or telephoning the practices. Ten interviews were carried out with 4 GPs, 3 research nurses, 3 members of practice management (1 interview with both a manager and a deputy manager) and 1 nurse practitioner (Table 2). Purposive sampling was used according to the demographic breakdown of recruiters and to ensure a range of participants based on practice recruitment levels and staff roles. Participants were recruited from practices with low (0–2 patients recruited), medium (3 to 4 patients recruited) and high (5 or more patients recruited) recruitment levels. This distinction was an arbitrary one and based on researcher experience. Participants were also recruited with both clinical and managerial roles.

**Table 2:** Demographic breakdown of recruiters

ID number	Gender	Occupation	Type of recruiter	Number of patients recruited at time of interview
(1)	M	General practitioner	Low	1
(2)	F	Research nurse	High	7
(3)	F	Research nurse	High	5
(4)	F	Practice manager	Medium	3
(5)	F	Research nurse	Low	2
(6)	M + F	Practice manager + deputy practice manager	Low	0
(7)	F	General practitioner	High	6
(8)	M	General practitioner	Low	0
(9)	F	General practitioner	Medium	4
(10)	F	Nurse practitioner	High	12

### The qualitative interviews

The trial and the interviews were carried out simultaneously. The audio-recorded interviews, which lasted between 22 min and 46 min, were carried out by the first author of this paper (HP), a female researcher with a good knowledge of the healthcare system and experienced in qualitative interviewing. The researcher had a clinical background but assumed the researcher role for the interviews. She did not hold any strong views about lung disease diagnosis trials and remained neutral on issues that were discussed with the recruiters. The interviews were carried out at the participants’ place of work, the general practices with only interviewees being present.

The interview guide reflected the aim of the study: to explore the feasibility of individually randomising patients to an urgent chest X-ray or not and to investigate any barriers to patient recruitment and participation. Topics included the following:

- Feelings about participating in a trial concerning lung disease
- Reasons for taking part in the trial
- Experiences of taking part in the trial (including practice training to participate in the trial, the use of trial documentation, the recruitment of patients, the referral process)
- Motivators/demotivators in participating in the trial
- Thoughts on the equipoise of the trial

No new topics emerged during the first few interviews so the interview guide remained unchanged. Field notes were carried out by the qualitative researcher immediately following the interview.

### Data analysis

Staff interviews were uploaded via digital media for transcription using a standard operating procedure (SOP) to ensure participant confidentiality. These were then transcribed verbatim and anonymised before being uploaded to NVivo 10 software [23] where relevant extracts were isolated and coded. Data analysis was conducted using Framework Analysis [24]. This analysis technique includes familiarisation (where the researcher becomes immersed in the data), developing a theoretical framework (where a hierarchical thematic framework is developed to classify and organise data into key themes, concepts, and categories), indexing (where the framework is applied to the original data transcripts and coded accordingly) and charting (where each theme is charted using a table or matrix using summaries of the data) and mapping and interpretation (where the charts and data are examined for patterns and connections). The main qualitative researcher was assisted by a second qualitative researcher who carried out 10% of the interviews to ensure validity of the analysis and to verify interpretation. A thematic hierarchy was produced and any disagreements were resolved through discussion.

## Results

These results reflect the aim of the study and the topics set out in the interview guide. They highlight what motivated practices to participate in the trial as recruiters, how practice staff interacted with patients recruited onto the study, what methods of organisation the practice used to undertake the trial, the general impact of the trial on practice staff, how the trial research team supported the practices and lastly the practice staff suggestions for trial delivery improvement. Table 3 summarises the results of our study, and Table 4 suggests actions to assist patient recruitment into a lung diagnosis trial.

**Table 3:** Study findings/themes

**Practice ‘motivators’ to join the trial as recruiters**	
• Having an existing interest in the research topic • Having the required eligible patient population registered at the practice • Being financially reimbursed • Improving lung cancer diagnosis • Developing the practice • Expanding the practice network • Personal and professional development of staff (having clinical and research learning opportunities) • Having altruistic, benevolent feelings	
**Health professional interactions with patients**	
*Challenges of identifying and recruiting eligible patients* • Patient eligibility criteria deemed too narrow • Staff fears that patients would have unnecessary chest X-rays • GPs feeling overworked and too busy to recruit • Having a lack of staff to participate in recruitment • Eligible patients being too busy to participate • Recruitment documentation confusing resulting in embarrassment and a reluctance to recruit • GPs too busy dealing with patients’ other needs	
*Presenting the trial to patients* • Staff worries that trial would increase patient anxiety about lung cancer (resulting in staff carefully choosing words to explain trial purpose) • The popularity of advertising chest X-rays (*at the time*) reduced staff worries of increasing patient anxiety • Ex-smokers were deemed easier to recruit	
*Completion of patient questionnaires* • Certain questions were deemed irrelevant to study • Certain questions were deemed too confidential for staff other than GPs to ask • Many patients required assistance to populate health questionnaires. • Health questionnaires taking too long to complete • Staff lacking time to help patients complete health questionnaires	
*Explaining chest X-ray referrals to patients* • Staff admitted they had mistaken perceptions that patients would prefer randomisation to the chest X-ray rather than control arm of trial • Patients not having a preference for participation in particular arm of trial was attributed to them being well informed of study equipoise • A minority of patients were deemed to feel anxious waiting for chest X-ray results	
**Practice organisation to undertake trial**	
*The importance of establishing key staff members responsible for the study* • One staff member is required to be accountable for the study • Continuity of staff is necessary. (A reliance on GP locums does not help the trial conduct) • Part time staff struggle to manage trial commitment • Research nurses who are paid especially to undertake trials are important *The need for a comprehensive recruitment system* • A team effort is required to recruit patients • A clear plan is necessary to recruit patients • Recruitment database searches require timely action to avoid losing potential patient recruits *Organisation of chest X-ray referral* • Patient feedback to staff highlighted easy travel to hospitals whilst others complained of travel distance or no free parking • Patient feedback to staff highlighted easy and efficient access to chest X-rays • Staff contact with X-rays Departments highlighted the X-ray staff being unaware of the study	
**General impact of trial on practice staff** • Staff more aware of long waiting times for a cancer diagnosis • Staff increased their knowledge of chest X-ray safety • Staff increased their knowledge of research methods and research engagement • Trial required one high recruiting practice to increase hours of practice nurse and take on a locum nurse • Staff anxiety due to concern for patients waiting for chest X-ray results • Staff fears of invoking unnecessary anxiety in patients	
**Researcher team support and involvement** *Impact of the research team* • Staff happy with involvement and support of research team • Staff felt that newsletter from research team were beneficial and helped them focus on the trial *Training day delivered by the research team* • Research team helped to reassure practice staff • Training pitched at a level for non-clinicians beneficial • Staff found literature given out beneficial before start of trial • Training was too detailed with too much paperwork leaving some staff feeling overwhelmed	
**Practice staff suggestions for improvements** • More administrative support • Decrease study reimbursement in exchange for assistance with recruitment • Share workload between research team and practice staff • Having practice managers and non-clinical workers consulted on trial design and delivery before start of trial • For practices to be given a mentor

**Table 4:** Suggested actions to assist patient recruitment into a lung diagnosis trial

**Practice ‘motivators’ to join the trial as recruiters**	
• The benefits to practice participation should be highlighted to practices. These can include financial reimbursement, CPD points or certificates for staff, gaining practice research accreditation status and clinical and research learning opportunities for staff • Initial discussions should focus on the practice’s clinical and research interests to determine practice focus • The numbers of required eligible patients registered at a Practice should be explored to scope for recruitment ability • The benefits to patients should be highlighted	
**Health professional interactions with patients**	
*The challenges of identifying and recruiting eligible patients* • Frequent discussions with recruiting staff should be employed at outset of study to feedback any problems • The benefits of research nurses to specifically recruit participants should be highlighted along with the possibility of them being financed by government bodies • Trial practice training days should offer evidence based information on the safety of chest X-rays • Trial practice training days should determine staff understanding of recruitment documentation	
*Presenting the trial to patients* • Advise staff on the use of non-anxiety invoking language with participants when discussing lung diagnosis	
*Completion of patient questionnaires* • Trial practice training days should check staff satisfaction with trial documentation • Practice staff should determine the staff member(s) who are best able to help populate participant documentation	
*Explaining chest X-ray referrals to patients* • Staff should focus on trial equipoise when discussing trial arms with patients • Advise practice staff that a small amount of patients may feel anxious when waiting for chest X-ray results	
**Practice organisation to undertake trial**	
• Encourage practice to choose one staff member who will be accountable for the trial • Advise practice staff of the efficacy of using only permanently employed staff where possible to better ensure continuity • Discuss with practice the possibility of using Government funded research nurses to engage with study documentation • Assist staff to have a clear recruitment plan before commencing trial	
**Researcher team support and involvement** • Research team to ensure frequent contact with practice staff at start and during the course of the study to determine the occurrence of any problems. Embedded qualitative research interviews with staff may highlight any challenges. • Research team to send regular newsletters to staff to ensure their continued focus on trial. • Great emphasis needs to be placed on practice training day to allow for discussion and questions. • Ensure practice staff (including non-clinical staff) are consulted on trial design and delivery before start of trial • Suggest to practices the possibility of engaging with a research practice mentor, one who has experience in participating in trials.	

### Practice motivation to join the trial as recruiters (Table 5)

#### A genuine interest in the trial focus

Recruiters’ motivation to take part in the study was largely due to a genuine interest in the focus of the ELCID clinical trial, which was to examine the effect on lung cancer diagnosis of lowering the threshold for referral for urgent chest X-ray for smokers and recent ex-smokers, aged over 60 with new chest symptoms.

**Table 5:** Practice motivation to join the trial as recruiters

A genuine interest in the trial focus	it’s something they were all very keen on doing because again of the potential impact that something like this might have if it goes undetected. (Recruiter 6, Practice Manager, Low Recruiter)
Having the required patient population	the decision to participate is more based on it being feasible to implement within the practice and if it’s a study we think we don’t have the patient population for, then we would be honest and open about that and say that we don’t think we could reach the target (Recruiter 5, Research Nurse, Low Recruiter)
Professional and practice development	we want to pick up more cancer patients (Recruiter 8, General Practitioner, Low Recruiter)
“	I think you know as a practice we’re always quite keen to sort of work with organisations such as yourselves to take these things forward (Recruiter 6, Practice Manager, Low Recruiter)
“	being part of “continuous professional development.” (Recruiter 2, Research Nurse, High Recruiter).
“	showing that you are ‘aware of N.I.C.E. guidelines’ (Recruiter 9, General Practitioner, Medium Recruiter)
“	taking part in the study is advantageous for appraisals, ‘I’ve got revalidation coming up. It’ll be mentioned’ (Recruiter 7, general practitioner, high recruiter).
The ‘feel good’ factor	to “improve medical care”, “do their bit” and “feel good about it after they’ve done it” (Recruiter 3, Research Nurse, High Recruiter).
The financial incentive	if there hadn’t been any (financial incentive) [...] we wouldn’t have engaged. (Recruiter 8 General Practitioner, Low Recruiter)
“	as a business we have to look to in terms of whether or not it’s financially viable for the practice to do it (the trial) and what the workload implications are. (Recruiter 6 Practice Manager, Low Recruiter)
“	it’s provided additional revenue [...] um for the surgery which has allowed us to employ more staff, um it’s had a number of indirect benefits to the practice as well. (Recruiter 5 Practice Manager, Low Recruiter)
“	study payment paying for staff a risk: ‘you don’t know how well it’s gonna work and how many people you’re gonna recruit’ (Recruiter 9 General Practitioner, Medium Recruiter)

#### Having the required patient population

Many also said that they took part because they believed that they had the appropriate patient population.

#### Professional and practice development

An additional motivation was the need to develop the practice and improve cancer diagnosis. Others talked of the desire to develop professionally in an individual capacity, for example, it would help with their personal profile and enable them to document the study. Participating in the study was also deemed as evidence of awareness of the NICE guidelines as well as it being advantageous for appraisals. The desire to expand the surgery’s network through making connections with research institutions was also highlighted.

#### The ‘feel-good’ factor

The feel-good factor of why staff participated in the trial was also highlighted.

#### The financial incentive

The importance of the financial incentive in relation to trial engagement was hailed as being significant to the extent that participation in the study would have been at stake. It was highlighted that a GP surgery is run as a business which has staffing implications, hence the importance of finance. The financial incentive had allowed one surgery to expand. Similarly, one GP pointed out that if they recruited enough patients onto the trial, then the two healthcare assistants that helped with recruitment would pay for themselves.

### Health professionals’ interactions with patients (Table 6)

This theme captured recruiters’ experiences of the recruiting process: problems identifying eligible patients, presenting the trial to the patients, referring patients for chest X-rays and providing patient support.

**Table 6:** Health professionals’ interactions with patients

Problems and disincentives to identifying eligible patients	Few patients were being recruited: Thirteen patients and six people come in with a chest infection [...] So just look at those figures, we look at half the people that come to our sit and wait surgery for an acute problem, about half of them are coming with a chest infection [...] well it does raise a question, why isn’t ELCID trial, why aren’t they being referred? [...] I don’t know the answer to that question. (Recruiter 4, practice manager, medium recruiter)
“	Eligibility criteria a problem: I didn’t end up identifying any of them I thought would fit the trial […] I don’t think we actually have as many patients who do qualify as we, we thought (Recruiter 8, general practitioner, low recruiter)
“	Eligibility criteria a problem: There’s a lot of patients I could have (recruited), if that (the eligibility criteria) had been a little bit more flexible, (Recruiter 1, general practitioner, low recruiter)
“	Unnecessary treatment causing fear: “unnecessary x-rays” (Recruiter 8, General Practitioner, Low Recruiter
“	Too much work: ‘it did sound very um complex and labour intensive’ (Recruiter 7, General Practitioner, High Recruiter).
“	Too busy: it’s ten past nine on a Monday morning, I’ve got thirty people waiting to be seen. Patient presents with an exacerbation of COPD and then you sort of... actually, I’ll do [recruit] the next one. (Recruiter 1, General Practitioner, Low Recruiter)
“	Study confusion may highlight lack of study knowledge: exposes my, my ignorance about what I’m supposed to be doing [...] be doing next. (Recruiter 8 General Practitioner, Low Recruiter)
“	Indifference to the trial: ‘you know, apathy just disinclination’ (Recruiter 8, General Practitioner, Low Recruiter).
“	Patients too busy: If they’re just popping in for a quick appointment and have to get off to work or got other commitments, then they’re less likely to want to participate. (Recruiter 5, Research Nurse, Low Recruiter)
“	Financial constraints: There’s no money in the health service at the moment and […] we’ve been told about prescribing and referring people in for unnecessary scans, x-rays whatever […] and I think there may be almost that subconscious element is oh, do I really need to refer this patient for an x-ray. (Recruiter 6, Practice Manager, Low Recruiter)
Presenting the trial to patients	Patients with other needs or pressed for time: sometimes we’re dealing with patients who are elderly, so can’t hear very well [...] who perhaps have got an appointment in the hair dressers in twenty minutes (Recruiter 4 Practice Manager, Medium Recruiter)
“	Easier to recruit ex-smokers: it’s very natural then to say well as you’ve been a smoker, very glad you’ve stopped, but as you’ve been a smoker you would be eligible for this trial. (Recruiter 7, General Practitioner, High Recruiter)
“	Fear of worrying patients: might give them the wrong signal and they may read something in to it that’s not there. (Recruiter 6, Practice Manager, Low Recruiter)
“	Methods to allay patient anxiety: probably don’t say the C word until quite a way in to the explanation (Recruiter 5, Research Nurse, Low Recruiter)
“	Telling patients the study is to change research and practice: but it’s to help research. [I] explain why we’re doing it because um, not because I think they’ve got lung cancer but because I, I would be doing other things if I thought that [...] But we want to improve our diagnostic skills and most people are very positive. (Recruiter 7, General Practitioner, High Recruiter)
“	Telling patients the study is to change research and practice: normally we wouldn’t send you for an x-ray at this stage, but this study is particularly looking to see if we should change that practice. (Recruiter 9, General Practitioner, Medium Recruiter)
“	Chest x-rays currently popular anyway: I think some of the messages in terms of the advertising campaigns about chest x-ray patients will... increasingly we find patients are bringing that up [...] Earlier chest x-rays. So they’re happy to have a chest x-ray [...] generally. I don’t think it caused particular anxiety. (Recruiter 3, Research Nurse, High Recruiter)
Completion of patient health questionnaires	Lengthy documentation: “onerous.” (Recruiter 1 General Practitioner, Low Recruiter) took “the best part of thirty minutes [to complete]” (Recruiter 4 Practice Manager, Medium Recruiter).
“	No problem helping patients to populate questionnaires: “quite straight forward” and unchallenging (Recruiter 5, Research Nurse, Low Recruiter).
“	Irrelevant study questions requiring assistance: ‘not relevant to the study’ ‘needed assistance completing them (Recruiter 4, Practice Manager, Medium Recruiter)
“	Better that the General Practitioner asks the questions: when a patient comes in and it’s the doctor, they trust that doctor. (Recruiter 4, Practice Manager, Medium Recruiter)
“	Unprepared and untrained to discuss confidential information: It’s awful for the patient to tell a member of staff that they know, we work in the reception area. I don’t say I’m a manager, you know I’m one of the girls [...] You know I’m telling the receptionist how depressed I’ve been feeling. It doesn’t seem professional. (Recruiter 4, Practice Manager, Medium Recruiter)
“	Discomfort and irrelevance of some questions: they’re happy to tell you about what their symptoms are and what medication they’ve been on and have they seen the pharmacist and the other things [...] but they look at you a bit strange [if you ask them depression screening questions] (Recruiter 2, Research Nurse, High Recruiter
Explaining study chest X-ray referral to patients	Patients happy to have chest X-ray or not: Everybody thought that you know these patients are going to feel cheated if they don’t get a chest x-ray but in reality, I’ve actually found it the other way round […] you know they’re feeling so poorly that they don’t really feel like going up to (hospital name) for a chest X-ray so that’s been quite a surprise. (Recruiter 3, Research Nurse, High Recruiter)
“	Patient reassurance about having a chest X-ray if needed: ... if you’re not randomised to have a chest x-ray and your symptoms persist or become worse then you would come back to see a clinician as you would do normally and a decision would be made whether to send you for a chest x-ray at that point. So you’re not going to be deprived of a particular treatment. (Recruiter 5, Research Nurse, Low Recruiter)
Patient feedback to health professionals following recruitment	Additional investigations following chest X-ray causing anxiety: [she was] extremely worried for the two to three week period that it took for the CT scan even though it was an urgent request. (Recruiter 5, Research Nurse, Low Recruiter)

#### Problems and disincentives to identifying eligible patients

Although patients with chest infections were presenting at the practices regularly, few patients were being recruited. Recruiting patients onto the trial was a problem for the practice staff. Difficulties recruiting were subsequently highlighted with reports of the eligibility criteria being too narrow as well as overestimation of how many practice patients would fit the eligibility criteria. Ultimately, the eligibility criteria had to be revised. Disincentives to identifying patients for the research study included the fear of patients having unnecessary X-rays and the fear of GPs being overworked and too busy. Confusion surrounding the whole study process led to staff not wanting to identify any patients, as it highlighted to others confusion surrounding the study process. A general indifference to the task of trial recruitment was also noted. Patient lack of time was also proposed if patients were busy. Financial constraints were also highlighted as demotivators to recruit patients.

#### Presenting the trial to patients

The GPs most commonly had first contact with patients regarding the trial and the research discussion would take place during the consultation. The patient would usually then be referred to another health professional for further information.

It was stated that the study was difficult to carry out if patients had other needs or were pressed for time although recruiting an ex-smoker for a lung diagnosis trial was deemed easy during a consultation. Many practices telephoned patients who may have been eligible for the trial. However, worries about increasing a patient’s anxiety by telephoning them to discuss the study were highlighted. Several methods were used to allay patient anxiety such as choosing one’s language carefully or explaining to the patient that they were not looking for cancer, but rather wanted to help research or change practice. However, most of the recruiters said that patient anxiety had not been a problem because of the current popularity of advertising early chest X-rays.

#### Completion of patient health questionnaires

Documentation presented a problem because of the lengthy time it took to finish. Helping patients to populate the patient questionnaires was unproblematic for other recruiters. However, one recruiter in particular, highlighted several problems with them for example, some questions not being relevant to the study and patients not understanding the questions and needing help to populate the questionnaires. This same recruiter stated that it is the GP who should be asking the questions to better reassure the patient and felt unprepared and untrained for hearing confidential patient information. Similarly, this awkwardness with asking patients personal questions was reiterated along with querying the relevance of some of the questions.

#### Explaining study chest X-ray referral to patients

Recruiters initially thought that all patients would want a chest X-ray referral on agreeing to participate in the trial. However, they discovered that patients either did not mind if they received a chest X-ray or preferred not to have a chest X-ray. Patients were reassured however that they would not be deprived of having a chest X-ray if the GP felt that it was necessary.

#### Patient feedback to health professionals following recruitment

Two recruiters shared feedback on the impact that the trial had on patients’ wellbeing during the trial. They stated that if the patients received a chest X-ray, then they were more likely to be anxious or worried in the period it took to receive their results. For example, a patient came back to the GP Practice following her chest X-ray to ask for her results which she had been worrying about. Moreover, another patient was anxious as following the chest X-ray; she had required additional investigations by way of a CT scan and had to wait for the results which ultimately were fine.

### Practice organisation to undertake the trial (Table 7)

To deal with these administrative challenges of running a study, increasing the hours of their practice nurse to compensate for the time it took to fill out the questionnaires with the patients was proposed.

**Table 7:** Practice organisation to undertake the trial

Establishing key staff members responsible for the study to ensure continuity	Having one person responsible for the trial: one of the biggest keys is having one um individual that is accountable um and is responsible for driving the research activity within the practice because without that it just flounders really and is never at the top of anybody’s priority list because there’s so much else going on. (Recruiter 5 Research Nurse, Low Recruiter)
“	Having one person responsible for the trial: I think if you haven’t got a dedicated person with set hours it would be very difficult [to run a trial.] (Recruiter 2 Research Nurse, High Recruiter)
“	Locum General Practitioners did not help with trial conduct: I don’t think that’s helped us as a practice in terms of stability and continuity so I think that’s probably hindered our opportunities to recruit. (Recruiter 6 Practice Manager, Low Recruiter)
“	Increased work load for General Practitioners if part-time Administrators did not work on the trial: “this cascade of additional work……..” which “defeated us.” (Recruiter 8 General Practitioner, Low Recruiter).
“	Importance of dedicated research nurse time: you know that’s [the trial is] what my dedicated hours are for and I think if that wasn’t the case then it would never get off the ground because they (the doctors listed as the principal investigators) are busy doing other things, you know? (Recruiter 2 Research Nurse, High Recruiter)
A team effort and a comprehensive recruitment system	Timely and prompt patient search required: if you leave it too long and look at it retrospectively you’ve... they may have been in and not got better and they’ll be back in again and then they’re already going to have a chest x-ray on their second visit possibly. (Recruiter 6 Practice Manager, Low Recruiter)
Organisation of the chest X-ray referral	Easily manageable for patients: we send them to the same hospital for the chest x-rays and it is local, so I wouldn’t have thought [...] they would’ve had any problems in getting there. (Recruiter 10 Nurse Practitioner, High Recruiter)
“	Chest X-rays accessible for patients: they’ve (the x-ray department) been really accommodating as well and would see the patients on the same day if we’d wanted to. (Recruiter 5 Research Nurse, Low Recruiter)
“	Chest X-rays accessible for patients: (The x-ray department offers) open access for anybody to have a chest x-ray so we just tell them (the recruited patients) to go down to (hospital name) one afternoon or one morning when it suits you. (Recruiter 2 Research Nurse, High)
“	Difficulty getting an X-ray: She was looking at maybe two week’s time and I said it is supposed to be urgent and she checked with someone and they said no, it’s not urgent. (Recruiter 3 Research Nurse, High Recruiter)
“	Problems with X-ray department being unaware of study: the radiologist rang me and said they weren’t aware of this study. (Recruiter 7 General Practitioner, High Recruiter) somebody was sent to the hospital and then they didn’t know anything about the study. (Recruiter 8 General Practitioner, Low Recruiter)
“	Difficulty accessing X-ray results: I couldn’t find the result anywhere and I had to find it on clinical portal (a Welsh, NHS digital patient information sharing platform). (Recruiter 3 Research Nurse, High Recruiter)

#### Establishing key staff members responsible for the study to ensure continuity

It was suggested that it was essential to have one person responsible for the trial in the practice to ensure its success. The importance of the continuity of staff working on the trial was also stressed. One practice had had to use locum GPs for 6 months which had not helped the conduct of the trial. Indeed, another practice decided that it would be GPs who worked full time that would work on the study whereas the administration team would not. This was because the latter worked part time hours and would not be able to manage the commitment. This, however, created a lot more work for the GPs. However, a research nurse pointed out that her role is specifically to do research since the GPs at the practice are too busy.

#### A team effort and a comprehensive recruitment system

Recruiters also discussed the necessity for a clear system to recruit patients including a comprehensive plan for recruiting patients which required a team effort. This practice manager would send a message to the GPs to remind them of the trial eligibility criteria in addition to posting the criteria on their computer screens. If the GP found a suitable patient, the patient was referred to a healthcare assistant or a research nurse for an explanation of the study. In addition, the recruiter also used an online system to check other patients in the practice. If eligible patients were found, the recruiter would call them to ask them to come to the practice. The need to be timely and prompt when recruiting patients retrospectively (searching for patients through practice database lists) was also proposed.

#### Organisation of the Chest X-ray referral

The recruiters discussed the logistics of patients getting to the hospital for a chest X-ray. Many said that it was an easy trip that the patients could manage. Others however said that it was more difficult as there was a lack of free parking or it was too far to the hospital. Others still talked of paying for a taxi for patients if necessary.

Patients were reported to have been able to access chest X-rays quickly and efficiently. Only one recruiter said that it was difficult to book an ‘urgent’ X-ray and stated that a patient could not get an X-ray appointment for 8–10 days. However, there were also some initial problems of the X-ray department being unaware of the study and accessing the X-ray results.

### General impact of the trial on the practice staff (Table 8)

The trial was seen to impact on the level of work which had to be conducted within the practice. A GP from a ‘high’ recruiting practice said that they had had to increase the hours of one of their practice Nurses and also take on a locum nurse. Another level of impact included practice staff anxiety due to waiting for patient chest X-ray results and concern for the patient waiting for the outcome. It was reported that a patient had become very anxious when they were being referred for a chest X-ray, and although the X-ray results were found to be clear, the clinician still felt guilty about invoking unnecessary anxiety for the patient.

**Table 8:** General impact of the trial on the practice staff

Increased staff time needed to carry out study: anybody who knows anything about medical research knows that there are quite a lot of hoops to jump through and you know, you have to dot the I’s and cross the T’s [...], it’s a commitment. (Recruiter 7 General Practitioner, High Recruiter)	
Staff concern for patients who had chest X-rays: everybody was a little bit aw, I hope she... I hope it’s ok, you know it’s sort of er ... um, that sort of feeling […] it’s a bit of a balancing act really on um you know sort of the pros and cons of the study and the pros and cons of patients participating as well. (Recruiter 5 Research Nurse, Low Recruiter)	
Feeling of guilt for unnecessary patient anxiety: I feel guilty now because I wouldn’t have sent her for a chest X-ray unless we were doing the study. (Recruiter 5 Research Nurse, Low Recruiter)	
Increase in medical knowledge: ‘we see so many different types of cancer and I don’t think I was aware that our diagnostic, you know the timing of our diagnosis was so delayed in this country. (Recruiter 7 General Practitioner, High Recruiter) I used to believe chest X-rays were dangerous things […] they’ve become a lot safer, so, it’s part of the mind-set anyway that we perhaps should be X-raying more people than we traditionally used to do […] certainly N.I.C.E. say that we should (Recruiter 8 General Practitioner, Low Recruiter)	
Increase in research knowledge: it’s an introduction into research for the, the practice and erm [...] And (Doctor’s name) has been pleased with how it has [...] turned out and so [...] maybe it’s a chance for of them stepping forward and taking more on [...] ‘Coz I know some practices they have um special research nurses there. (Recruiter 10 Nurse Practitioner, High Recruiter)	

However, the most common impact of the trial on practice staff was an increase in medical knowledge for example the length of delay in a possible cancer diagnosis. Another example is knowledge update on the safety of chest X-rays. An additional benefit of the trial related to knowledge of and engagement in research and research methods.

### Researcher team support and involvement (Table 9)

#### Input from the research team

The training day presented by the research team was deemed as being generally very good with the information being presented concisely and appropriately for the audience variety. Some though found the trial detail overwhelming which necessitated too much paperwork. However, the research nurse who had undergone Good Clinical Practice (GCP) training (whilst her two GP colleagues had not) thought she felt more comfortable in the training.

**Table 9:** Researcher team support and involvement

Input from research team	Training day generally good: we’d had a lot of the literature beforehand so we were more or less up to speed with what we need to do before the training session but it’s always nice to have it reinforced. (Recruiter 5 Research Nurse, Low Recruiter) (the presentation) was very good and it was pitched at a level where we as non-clinicians could understand it. (Recruiter 6 Practice Manager, Low Recruiter)
“	Trial detail overwhelming: I just wonder whether we were trying to do too much. There’s too much detail. (Recruiter 1 General Practitioner, Low Recruiter) A lot of paperwork: I do remember thinking it’s an awful lot of [...] paperwork. (Recruiter 8 General Practitioner, Low Recruiter)
“	Easier for those who had undertaken GCP training: I think they [the General Practitioners] found it a bit more overwhelming than I did. (Recruiter 2 Research Nurse, High Recruiter)
“	On-going study team support helpful: ‘to keep us focused’ (Recruiter 2 Research Nurse, High Recruiter
“	Newsletters aiding patient recruitment: it gives you that... that little bit of a competitive edge on um, on your recruitment if you can see that other practices are doing well or um tips on what they are doing that perhaps you might not be doing. (Recruiter 5 Research Nurse, Low Recruiter)
“	Genial nature of research team: helpful we’ve had a lot of support from the team. We’ve had newsletters. She (the trial manager) rung and emailed us to ask if there’s anything she can help with. (Recruiter 6 Practice Manager, Low Recruiter) She (the trial manager) emailed me a couple of times and I’ve... if I’ve had a question, she’s very good and she emails you back straight away you know [...] you feel quite supported. (Recruiter 2 Research Nurse, High Recruiter)

Regarding ongoing support from the study team, the newsletters were described as helpful*.* Others highlighted the competitive nature of the newsletters which had a positive impact on the practice recruitment activity. The newsletters were also purported to be reassuring as recruiters could see that other practices were struggling to recruit also.

The genial nature of the research team was also highlighted by the practice staff, for example with regard to the support and encouragement that they received during the course of the trial.

### Practice staff suggestions for improvement (Table 10)

The most popular suggestion for study improvement was the request for more administrative support. There was a proposal for ‘a workload share’ between the practice staff and the research study team, where the practice staff could scan patient records for eligibility whilst the research study team could explain the study to potential participants. It was even pointed out that the practice would be happy to decrease their study reimbursement if they had someone to help with recruitment. It was acknowledged though that this strategy may not be the best use of resources for the research study as recruitment numbers could be minimal in each individual practice. Furthermore, a nurse recruiter stated that since it is a GP alone that could consent patients, the research study team was limited in helping to conduct the study anyway*.*

**Table 10:** Practice staff suggestions for improvement

More input from research team required: if all the admin [...] apart from identifying the patient [...] and arranging the chest x-ray, was taken out of our hands [...] then that would’ve definitely been easier. (Recruiter 8 General Practitioner, Low Recruiter)	
Preference for less study payment and more recruitment help: we’d be more than happy to you know, decrease the money if we had somebody here to help out with the recruitment. (Recruiter 1 General Practitioner, Low Recruiter)	
Problems with recruitment help: it (providing administrative support) wasn’t going to be worth their (the study team’s) whilst unless we could get about 3, a minimum of 3 patients [...] And we’re not enrolling at that sort of rate. (Recruiter 7 General Practitioner, High Recruiter)	
Importance of Practice Managers having input into study design: ‘needing in terms of the delivery of it (the trial), it’s almost a little like Ivory Tower thinking in that this is what we’re going to do and it’s easy to do it. And those individuals not having an understanding of what goes on in primary care. (Recruiter 6 Practice Manager, Low Recruiter)	
Having the support of a mentor: and supported with education and updates and things like that." (Recruiter 2 Research Nurse, High Recruiter).	

Suggestions on how the study team could help the practices in the future included the practice managers/non clinical workers being consulted on the design of the trial and delivery of the trial. Also, one participant thought it would be beneficial having the support of a mentor.

## Discussion

Our study focused on a lung diagnostic practice in general practice. It is the first to explore the experiences of general practice staff participating in and recruiting to a lung diagnostic trial. However, it also has relevance to the conduct of trials in primary care in general. Tables 3 and 4 set out the practical implications of the study findings. The following discussion reflects on the findings and their associations with existing literature.

### The recruitment challenges and enhanced workload associated with a lung diagnosis trial

The issue relating to a strict ELCID patient eligibility status (which consequently needed revising) has been reflected in other studies. In their systematic review on effective recruitment strategies in primary care research, Ngune and colleagues [25] suggested using simple patient eligibility criteria to enhance recruitment. Hange and colleagues [26] too emphasised the importance of scrutinising the inclusion criteria in detail to ensure relevance to practice. In spite of careful consideration to the inclusion and exclusion criteria at the protocol stage of the trial, patient recruitment was quickly identified as a problem and the qualitative interviews highlighted the strict eligibility criteria being the issue. The failure to recruit according to our initial eligibility criteria could be associated with the significant decline in the UK population smoking [27]. Future trials concerning lung cancer diagnosis may therefore need to accept participants who have given up smoking for longer than 5 years, in order to meet the required recruitment target numbers.

Another reason for poor recruitment into the ELCID trial could be due to the clinician’s busy workload. For example, a study clinician admitted to dissuading himself from recruiting a patient if his clinic was busy, and because of this, lacked the motivation to recruit. This could be why one of the practice managers of a low recruiting practice had difficulty in understanding the poor recruitment rates since she had noted many eligible patients to recruit. Time challenges were identified as reasons for reluctance to recruit participants into other primary care studies [4, 26, 28–30]. Hange and colleagues [26] highlighted the difficulty that GPs have combining research and clinical work and stress the complicated and time consuming organisational demands of enrolling patients. Foster and colleagues [4] too found that poor recruitment into a study was associated with longer times to recruit the first patients. It is noteworthy that the research nurses in our study had roles that were dedicated to doing research and were both located in the high recruitment bracket (Table 2). Similarly, Potter and colleagues [31] highlighted that the research nurses in their study who had dedicated research time were those who recruited more successfully. Our study also highlighted that assistance with patient documentation was found to be time consuming for some staff, as was the case with other studies [32, 33]. The ELCID staff also had an additional time burden of dealing with X-ray departments.

An additional reason for GP reluctance to recruit was possibly worry over giving unnecessary chest X-rays to patients. For example, one of the GPs stated that he had originally been worried about the safety of the chest X-rays but the ELCID trial training had updated his knowledge and relayed his fears. Other practice staff also exhibited worries that the study could cause patient anxieties being recruited onto a lung diagnostic study which could diagnose cancer. This issue however was largely unfounded as ELCID patient interviews highlighted only a small number of patients being anxious regarding their participation [14]. Staff worries concerning patient reluctance to participate in the study control group, that is, not having the chest X-ray could also have affected clinician recruitment. This worry proved unfounded since although patients stated that they did prefer to have the chest X-ray, they still agreed to participate in the study [14].

### Drivers to successful recruitment in a lung disease diagnosis trial

Understanding what motivates staff to recruit participants into trials has shown to be paramount when considering recruitment difficulties in primary care [6, 11, 34–36].

The ELCID staff interviews showed that a genuine interest in the topic of lung disease diagnosis helped to motivate staff to recruit. Several studies have shown similar links to improved recruitment in primary care, for example having a special interest in the trial subject area [37], the research question being of relevance [29, 38] and the benefits that the trial is providing [28, 39].

Our study also mirrors several studies with regard to the importance of being reimbursed financially [5, 30, 32, 40]. Several of our study GP practices used this financial incentive to pay for staff to recruit study participants. However, the fact that the most popular suggestion by the practices to improve the study was the request for more administrative support (with one even happy to decrease their financial reimbursement in return for help with recruitment) highlights how busy they were. It is noteworthy that the practice who recruited the most patients employed a research nurse to focus especially on research studies. This nurse had been funded by the Wales Primary Care Research Incentive Scheme (PiCRIS) [41] although this scheme is currently being revised [41]. Schemes like this are imperative if general practice is to continue to play an important role in improving patient care through research and innovation. This scheme also provides the research accreditation status that many staff desire.

Professional staff development and expanding the surgery’s research network as motivators to recruit in the ELCID study mirrored other studies, for example the importance of providing Continuing Professional Development (CPD) points [42], enjoying increased professional satisfaction [43] and having an interest in improving clinical practice [44].

### Optimum organisation and continuity of care in a lung diagnosis trial in general practice

In their systematic review of effective recruitment strategies in primary care research, Ngune and colleagues [25] highlighted concepts of good organisational practices. These included the involvement of a discipline champion, having simple patient eligibility criteria and using strategies that reduce practitioner workload. They also point to the active participation of the primary care staff in the design and conduct of the research to enable effective strategies specific to the context of care delivery.

Our ELCID study staff participants highlighted similar concepts including the suggestion of one specific staff member taking responsibility for the trial within each practice. The fact that the research nurses recruited the most participants suggests that they may be the best staff member to take on this role. Continuity of staff member dealing with patient may be a beneficial aspect when a GP practice carries out a lung diagnosis study. The study may require arranging chest X-rays for anxious patients who may need further tests. Moreover, continued communication with a patient may help non-clinical staff feel more comfortable discussing health questions with patients for data collection. This was a problem highlighted by one of the ELCID practice managers.

### Support and involvement by research team

Gaglio and colleagues [45] highlighted the importance of a strong rapport between researchers and practice staff in a primary care diabetes behaviour change programme and point out that this focus plays a key factor in research success. Our study staff too emphasised the genial nature of the study team and the good support that they received. Some staff found the study training day overwhelming, thus highlighting the necessity of continuing researcher support in trials. Our use of newsletters has also been used successfully in other studies to provide focus and reminders to staff [31, 46, 47].

### Strengths and limitations of the study

Our study was the first to explore general practice participation in a trial to detect possible lung disease. It also used embedded qualitative research methods to enhance learning associated with the trial. A cross section of the staff that were interviewed included practice managers, GPs, practice nurses, research nurses and administrative staff, and this allowed for a more complete picture of staff participation in the study. However, although the study interviewed a variety of staff that worked at the practice, numbers of staff interviewed per group were small. A larger number of interviews of each staff group would have generated a clearer picture of the attitudes and experiences of each staff group.

## Conclusion and recommendations

The integration of a qualitative component focused on staff experiences participating in a lung diagnostic trial has demonstrated the feasibility to recruit for similar future studies within general practice. Although recruitment into trials can be difficult, results from our study offer suggestions on maximising patient recruitment not just to trials in general but also specifically for a lung diagnosis study. Table 4 suggests key recommendations to maximise patient recruitment in general practice trials.

## Data Availability

Study data will always remain confidential whilst the study is ongoing. Once the study has been completed, the data has been ratified and results published, the data will be made available to external academic researchers. Requests for access to data will be directed to the Marie Curie Research Centre, where the Scientific Director of the Centre will be identified as the data custodian for the trial and will ensure adherence with Marie Curie and University policy. Where the study has been undertaken in conjunction with a Clinical Trials Unit, the request for data release will be made to that trials unit and processed in accordance with their data sharing protocols.
